# A critical moment for neonatology: appeals from the Lancet Child & Adolescent Health Commission

**DOI:** 10.1186/s13052-026-02214-9

**Published:** 2026-03-26

**Authors:** Carlo Dani, Giovanni Corsello, Daniele De Luca, Massimo Agosti

**Affiliations:** 1 Department of Neurosciences, Psychology, Drug Research and Child Health, University of Florence, Florence, Italy; 2 Division of Neonatology, Careggi University Hospital of Florence, Largo Brambilla 3, Florence, 50134 Italy; 3 Department of Health Promotion, Mother and Child Care, Internal Medicine and Medical Specialties “G. D’Alessandro”, University of Palermo, Palermo, Italy; 4 Division of Pediatrics and Neonatal Critical Care, A. Béclère Medical Center, Paris Saclay University Hospitals, Paris, France; 5 Department of Medicine and Technical Innovation, Hospital “F. Del Ponte”, University of Insubria, Pediatrics, Varese, Italy

## Abstract

**Background:**

The Lancet Child and Adolescent Health Committee on the Future of Neonatology has published an important document on the future of neonatology to highlight the significant challenges hindering the development of research in this field of medicine.

**Main body:**

The number of preclinical and clinical studies in neonatology is insufficient due to a lack of interest from industry and insufficient support from public agencies. In many countries, neonatology is not formally recognized as a medical specialty, which fosters a shortage of academic positions, with negative effects on both neonatology research and the training of young neonatologists. This situation is exacerbated by excessive bureaucracy, which hinders productive interaction with regulatory agencies and ethics committees and makes conducting randomized controlled clinical trials excessively expensive. The lack of shared standardized outcome definitions and the limited use of real-world data contribute to making neonatology research more difficult.

**Conclusion:**

We support the Commission’s proposal for a Global Alliance for Innovation in Neonatal Health (GAINH), which would include neonatologists, former patients, families, industry, and public bodies, such as governments and universities, to address and remove barriers to advancing neonatal health through high-impact research and innovation.

## Background

Last June, three years after its establishment, the Lancet Child & Adolescent Health Commission on the future of neonatology published an important document on the future of neonatology [1]. This commission was born as a reaction to the unexpected interruption of a multicentre, phase 2b trial of recombinant human insulin-like growth factor 1 (IGF1) for preventing bronchopulmonary dysplasia [2] decided by a pharmaceutical company without any regard for the patients and their families who had consented to participate in the study in light of the potential benefits. Although after a few years this study was restarted with the support of another pharmaceutical company, this event caused a great impression and raised several questions about the future of neonatology. It was recognized that over the past few decades, there has been little real progress in the treatment of newborns—particularly preterm infants—and that current therapies differ little from those used 20 or 30 years ago. This situation has arisen due to a variety of factors. The purpose of establishing the Commission is precisely to identify these factors and propose possible solutions. To this end, the Commission was intentionally composed of a multidisciplinary team, including neonatologists, public health specialists, pediatricians, pediatric surgeons, maternal and fetal medicine experts, nurses, developmental biologists, clinical pharmacologists, technologists, and representatives from regulatory bodies, patient and parent groups, and the World Health Organization (WHO) [1].

The commission had four main objectives: (1) highlight the causes that hinder the development of neonatology research; (2) emphasize how solid scientific knowledge is essential to improving neonatal medicine; (3) advocate for greater financial and cultural investment in neonatal research; and (4) provide recommendations to facilitate the development of new drugs and medical devices for critically ill newborns [1].

## Main text

The number of preclinical and clinical studies in neonatology is certainly insufficient. This is attributable both to a lack of interest from industry, which devotes far less funding to neonatology than to adult studies, and to insufficient attention and funding from public agencies. It is possible that the lack of interest from the industry depends on the extremely small number of potential patients who can hardly guarantee a significant economic return. On the other hand, when we talk about the social role of the industry, it should also be understood that a small part of the investments should be made in sectors that are necessary from the point of view of public health even if with a very limited economic return. The role of public bodies should differ from that of industry and be characterized by a commitment to supporting those areas of research most neglected by private investment, such as neonatology. In practice, however, this is not the case: even in publicly funded research calls, neonatology receives little attention. While neonatologists must, of course, strive to submit high-quality projects, it is also true that they are rarely involved in the scientific committees responsible for evaluating proposals and allocating funding. As a result, research projects in neonatology often have a lower chance of being approved, regardless of their intrinsic quality. This lack of representation on committees may, in turn, be linked to the limited number of academic positions in neonatology—a situation that is particularly evident in countries with low birth rates, such as Italy [3]. Moreover, in many countries neonatology is not formally recognised as a medical specialty, and this also favours a deficit of academic positions which have negative effects on research [3].

Over two-thirds of Cochrane meta-analyses in neonatology are inconclusive due to studies that are too small or methodologically inadequate [4]. Furthermore, there are many examples of neonatal drugs whose development was stopped despite promising preclinical or clinical results, as well as examples of failed neonatal device development. However, this suboptimal state of research in our field often results in treatments that rely more on best practices than on solid evidence. This situation arises not only from a lack of funding but also from excessive bureaucracy, which hinders productive interaction with regulatory agencies and ethics committees. For small and medium-sized enterprises, the planning and execution of randomized controlled clinical trials are often unsustainable from both organizational and economic perspectives, forcing companies to abandon their projects. Consequently, researchers are left to carry out non-profit studies, which frequently suffer from resource limitations and are generally not considered adequate by regulatory authorities. These combined factors create a vicious cycle that leads to stagnation in research and prevents meaningful progress in neonatal care.

The Commission, therefore, rightly recalls that clear and simple indications to ethical and regulatory bodies are needed to simplify the procedures (and the costs) required for the development of neonatal drugs and devices and ensure high-quality research [1]. For example, it is unacceptable that neonatal trials being rejected for administrative reasons and no scientific issues despite their original approval. In this regard, the Commission rightly argues that overly stringent regulations and procedures, designed to protect patients during experimental studies, do not protect them from the lack of innovation [1].

Another important point is the possibility of not limiting research to randomized controlled trials alone, but rather exploiting the widespread availability of high-quality, real-world data [5]. This option is favored by the ever-increasing use of electronic medical records, which generally allow for the rapid selection of large numbers of patients based on diagnoses and treatments. On the other hand, the use of these tools requires caution to protect patient privacy. Families should be informed in advance, preferably upon admission, that the information in the medical record may be used for research purposes. Furthermore, large observational studies conducted using this approach may help achieve a more precise identification of the different phenotypes of neonatal diseases, which remain largely understudied. For example, it is known that respiratory distress syndrome [6] and bronchopulmonary dysplasia exhibit distinct phenotypes [7], yet they are often treated as if they were single, uniform conditions. It is reasonable to assume that this could lead to treatments that are effective for certain phenotypes but not for others, ultimately resulting in therapies being deemed ineffective when, in fact, they might benefit specific subgroups of patients.

When planning a research study, an important issue is the definition of the clinical outcome. Indeed, if we consider only the definition of bronchopulmonary dysplasia, the reduction of which is one of the most common endpoints in neonatology research, at least three definitions easily come to mind, and we know that applying one or the other significantly changes its frequency [8]. The problem becomes even more complicated if the endpoint is neurodevelopment, which can be assessed with various methods at quite different ages [9]. On the other hand, we know that it would also be appropriate to evaluate the effects of neonatal pathologies in adulthood, making it even more difficult to define this outcome in relation to the risk of developing behavioral and psychiatric disorders [10, 11]. Trying to homogenize these results by standardizing their definition would certainly represent a major step forward for neonatology research, improving the quality of studies and the accuracy of results.

To improve the future of neonatology, the Commission’s document concludes with recommendations for industry, healthcare professionals, former patients, and family associations, as well as governments and universities.

Industry should commit to funding more projects to develop drugs and devices for neonatal pathologies. Strengthening ties with neonatologists most involved in research would be very helpful in this regard. On the other hand, it must be recognized that the costs of developing new drugs and devices are enormous, and therefore it is necessary to develop new forms of collaboration between industry and public bodies, as well as to employ other fundraising strategies, such as supporting charities and crowdfunding.

Neonatologists should develop high-quality clinical and preclinical research projects and strive to initiate multidisciplinary collaborations with other medical specialties. To develop new drugs, it is necessary to intensify the use of modern technologies, such as point-of-care tools, biobanks, and artificial intelligence, paying particular attention to the different phenotypes of the most common neonatal diseases. Neonatologists should play a leading role in conducting research and caring for healthy and pathological newborns, even when faced with challenges from industry and regulatory agencies.

Former patients, their families, and organizations should make their voices heard at every opportunity to demand the best care for newborns, including demanding quality research. In addition to the political world, they should also actively interact and collaborate with regulatory agencies, ethics committees and universities. A recent example of the usefulness of this support was the pressure exerted by parents and organizations on policymakers to make nirsevimab available for the prevention of respiratory syncytial virus disease in many areas of our country. In several Italian regions, the fruitful collaboration between neonatologists and families contributed to the free availability of this highly effective prophylaxis for all children in the first year of life during the epidemic season. The success was resounding: hospitalizations for bronchiolitis caused by respiratory syncytial virus were reduced by 80–90% [12]. Similarly, the availability of an effective therapy and the strong push from parent associations led to the development and diffusion of newborn screening for spinal muscular atrophy (SMA), initially in two Italian regions, Tuscany and Lazio, and subsequently in many other regions [13].

The role of governments and universities remains central. Unfortunately, there appears to be a lack of genuine political awareness regarding the importance of promoting neonatal health, as well as an insufficient understanding that a healthy newborn is the foundation of a healthy child and adult—and thus a crucial investment in a better and more sustainable society. Only through the development of such awareness can greater confidence in neonatal medicine emerge, along with the corresponding willingness to allocate the resources necessary to foster its growth and advancement. A greater number of full professors of neonatology would be necessary and useful to promote both research in the neonatology and better training of young neonatologists [3]. However, the lack of an autonomous specialization greatly limits this possibility [3]. On the other hand, it seems unlikely that the initiative of a single country can change the situation and therefore action at the European level seems to be the most effective, albeit difficult, solution.

## Conclusions

The Commission’s document concludes with a proposal for a Global Alliance for Innovation in Neonatal Health (GAINH), which would include neonatologists, former patients, families, industry, and public bodies to address and remove barriers to neonatal health progress through high-impact research and innovation [1]. This Alliance should address and seek to resolve the various problems identified by the Commission. Establishing a timetable for achieving the many goals is truly difficult, and it would be naive not to recognize the enormous challenges this entails. On the other hand, great credit must be given to the Commission for expressing what many neonatologists worldwide strongly believe: that newborns should truly be at the centre of both the present and the future, something that currently seems far from being achieved. It’s a cliché that when it comes to newborns, everyone are deeply touched, but when it comes to putting words into action, there’s always some reason to prioritize and invest in other groups of people. Disrupting this way of thinking and, above all, acting should be everyone’s commitment, especially those most aware of it, namely the members of this Alliance. The availability of a document that summarises the main issues that concern the future of neonatology is certainly an excellent starting point for improving the situation and for ensuring that each of us can contribute to this improvement as much as possible [1].

## Data Availability

Not applicable.

## Abbreviations

GAINH: Global Alliance for Innovation in Neonatal Health
IGF1: insulin-like growth factor 1
WHO: World health organization

## References

[1] De Luca D, Modi N, Davis P, Kusuda S, de Wildt SN, Keszler M, et al. The lancet child & adolescent health commission on the future of neonatology. Lancet Child Adolesc Health. 2025;9:578–612. 40580970 10.1016/S2352-4642(25)00106-3

[2] Ley D, Hallberg B, Hansen-Pupp I, Cani C, Ramenghi LA, Marlow N, et al. rhIGF-1/rhIGFBP-3 in preterm infants: a phase 2 randomized controlled trial. J Pediatr. 2019;206:56–65. 30471715 10.1016/j.jpeds.2018.10.033 PMC6389415

[3] Sanchez-Luna M, Raimondi F, De Luca D. Neonatal professorships in Europe: insufficient to protect the tiniest and frailest. Lancet Reg Health Eur. 2025;50:101224. 39975585 10.1016/j.lanepe.2025.101224 PMC11835632

[4] Varrio T, De Luca D, Kuitunen I. Evidence certainty in neonatology—a meta-epidemiological analysis of cochrane reviews. Eur J Pediatr. 2025;184:191. 39934466 10.1007/s00431-025-06023-w PMC11814034

[5] Liu F, Panagiotakos D. Real-world data: a brief review of the methods, applications, challenges and opportunities. BMC Med Res Methodol. 2022;22:287. 36335315 10.1186/s12874-022-01768-6 PMC9636688

[6] Laughon M, Allred EN, Bose C, O’Shea TM, Van Marter LJ, Ehrenkranz RA, et al. Patterns of respiratory disease during the first 2 postnatal weeks in extremely premature infants. Pediatrics. 2009;123:1124–31. 19336371 10.1542/peds.2008-0862 PMC2852187

[7] Shepherd EG, Clouse BJ, Hasenstab KA, Sitaram S, Malleske DT, Nelin LD, et al. Infant pulmonary function testing and phenotypes in severe bronchopulmonary dysplasia. Pediatrics. 2018;141:e20173350. 29622720 10.1542/peds.2017-3350

[8] Isayama T, Lee SK, Yang J, Lee D, Daspal S, Dunn M, et al. Revisiting the definition of bronchopulmonary dysplasia: effect of changing Panoply of respiratory support for preterm neonates. JAMA Pediatr. 2017;171:271–9. 28114678 10.1001/jamapediatrics.2016.4141

[9] Cirelli I, Bickle Graz M, Tolsa JF. Comparison of Griffiths-II and Bayley-II tests for the developmental assessment of high-risk infants. Infant Behav Dev. 2015;41:17–25. 26276119 10.1016/j.infbeh.2015.06.004

[10] Montagna A, Karolis V, Batalle D, et al. ADHD symptoms and their neurodevelopmental correlates in children born very preterm. PLoS ONE. 2020;15:e0224343. 32126073 10.1371/journal.pone.0224343 PMC7053718

[11] Hadaya L, Nosarti C. The Neurobiological correlates of cognitive outcomes in adolescence and adulthood following very preterm birth. Semin Fetal Neonatal Med. 2020;25:101117. 32451305 10.1016/j.siny.2020.101117

[12] Attaianese F, Trapani S, Agostiniani R, Ambrosino N, Bertolucci G, Biasci P, et al. Effectiveness of a targeted infant RSV immunization strategy (2024–2025): A multicenter matched case-control study in a high-surveillance setting. J Infect. 2025;91:106600. 40848988 10.1016/j.jinf.2025.106600

[13] Abiusi E, Vaisfeld A, Fiori S, Novelli A, Spartano S, Faggiano MV, et al. Experience of a 2-year spinal muscular atrophy NBS pilot study in italy: towards specific guidelines and standard operating procedures for the molecular diagnosis. J Med Genet. 2023;60:697–705. 36414255 10.1136/jmg-2022-108873

